# Receptor and transporter binding and activity profiles of albiflorin extracted from *Radix paeoniae Alba*

**DOI:** 10.1038/srep33793

**Published:** 2016-09-20

**Authors:** Zeng-liang Jin, Nana Gao, Weizhe Xu, Pingxiang Xu, Shuaiqi Li, Yuan-yuan Zheng, Ming Xue

**Affiliations:** 1 Department of Pharmacology, School of Basic Medical Sciences, Capital Medical University, Beijing, P. R. China; 2 Department of Central Laboratory, Hospital of Beijing Shijitan, Capital Medical University, Beijing, P. R. China; 3 Hospital of Beijing Xuanwu, Capital Medical University, Beijing, P. R. China

## Abstract

Albiflorin, a traditional Chinese herb, is a main component of *Radix paeoniae Alba*, which has been used for the treatment of depressive disorders since ancient times. However, the mechanism of the antidepressant effect of albiflorin is poorly understood. Thus, we explored the binding profile of albiflorin at neurotransmitter receptors and transporters. We also characterised the *in vivo* effect of albiflorin on monoaminergic systems by using microanalysis to determine the extracellular levels of serotonin (5-HT) and norepinephrine (NE) in the hypothalamus of freely moving rats administered albiflorin. We found that albiflorin inhibited the uptake of 5-HT and NE and displayed robust binding affinities for the transporters of both neurotransmitters. By contrast, albiflorin (10 μM) showed no significant affinity to a wide array of central nervous system receptors. The results of our *in vivo* microdialysis studies showed that administration of albiflorin (3.5, 7.0, 14.0 mg/kg) significantly increased extracellular concentrations of 5-HT and NE in the hypothalamus of freely moving rats. Overall, the current study showed that albiflorin is a novel 5-HT and NE reuptake inhibitor with high selectivity.

Depression, a serious emotional disorder with cognitive, autonomic nervous system and endocrine dysfunctions, has an estimated lifetime prevalence as high as 21% of the general population in some developed countries^1^. The antidepressants currently in use include tricyclic antidepressants, selective serotonin reuptake inhibitors (SSRIs), monoamine oxidase inhibitors and serotonin (5-HT) and norepinephrine (NE) reuptake inhibitors. All of these antidepressants exert their effects by increasing the levels of 5-HT, NE, or both with known limitations of slow onset and adverse effects. Therefore, there is a strong need for the development of antidepressants with more rapid onset and fewer adverse effects. Traditional herbs provide a prospective alternative in the treatment of depression, and there is growing interest in the therapeutic effects of natural products on mental disorders. In particular, the antidepressant effects of a variety of traditional Chinese medicines, such as St. John’s Wort, marinade root, gingko, valerian and areca seed, used individually or in a formulated prescription, have received a great amount of attention because these natural products show no or very few adverse effects^2^,^3^.

Albiflorin, a monoterpene glycoside, is a main component of *Radix paeoniae Alba*, which is often used in herbal medicines in China, Korea and Japan. *Radix paeoniae Alba*, commonly known as a peony, is the root from *Paeonia lactiflora* Pall, a member of the family Ranunculaceae. This root is a component of numerous traditional formulae, including Jiaweisinisan and Dang Gui Shao Yao San, prescribed for the treatment of depression-like disorders^4^. Among these components, monoterpene glycosides extracts, such as paeoniflorin and albiflorin, are usually described as the most important active components of the peony^5^. Previous studies have found that that short- and long-term administration of albiflorin produce antidepressant-like effects, as evidenced by decreases in the duration of immobility in forced-swim and tail-suspension tests in mice and the reversal of chronic unpredicted mild stress-induced inhibition of sucrose consumption in rats^4^. In addition, albiflorin produces significant antidepressant-like effects closely related to hippocampal 5-HT and NE increases and BDNF expression, which are similar to the effects of the classic antidepressant SSRIs^4^. However, the monoaminergic mechanism and the receptor target of albiflorin remain unclear.

The monoaminergic system is one of the most important targets in the pathophysiology and therapy of depression^6^,^7^. Monoamine reuptake inhibitors work by binding to their respective transporter proteins located presynaptically. This binding interaction induces an increase in the elimination time of the neurotransmitter cycle; thus, the extracellular concentration of neurotransmitters increases in the synaptic cleft. This increase in neurotransmitters in the cleft allows for increased downstream cellular signalling^8^. In the present study, we used a series of competition binding assays and transporter inhibition bioassays to determine the affinities of albiflorin to the rat and cloned human (h) transporters for the monoamines 5-HT, NE and dopamine (SERT, NET and DAT, respectively). To determine the selectivity of albiflorin, we evaluated the affinity of albiflorin at numerous other targets, including receptors for dopamine (D1, D_2_, D_3_, D_4_, D_5_), 5-HT (5-HT_1A_, 5-HT_1B_, 5-HT_1D_, 5-HT_2a_, 5-HT_2C_, 5-HT_3_, 5-HT_5A_, 5-HT_6_, 5-HT_7_,), NE (α_1A_, α_1B_, α_2A_, β_1_), acetylcholine (muscarinic M_1_, M_2_, M_3_, M_4_, M_5_), opioids (μ, κ, δ), adenosine (A_1_, A_2A_) and histamine (H_1_, H_2_, H_3_, H_4_). In addition, we tested the effect of albiflorin on the transport activities of the three monoamines using transporter-transfected HEK293 cell lines as well as a rat brain synaptosomal preparation. Furthermore, to characterise the *in vivo* effect of albiflorin on monoaminergic systems, we used microdialysis to determine the extracellular levels of 5-HT and NE in the hypothalamus of freely moving rats administered albiflorin.

## Results

### Binding of albiflorin to rat monoamine transporters

**Figure 1:**
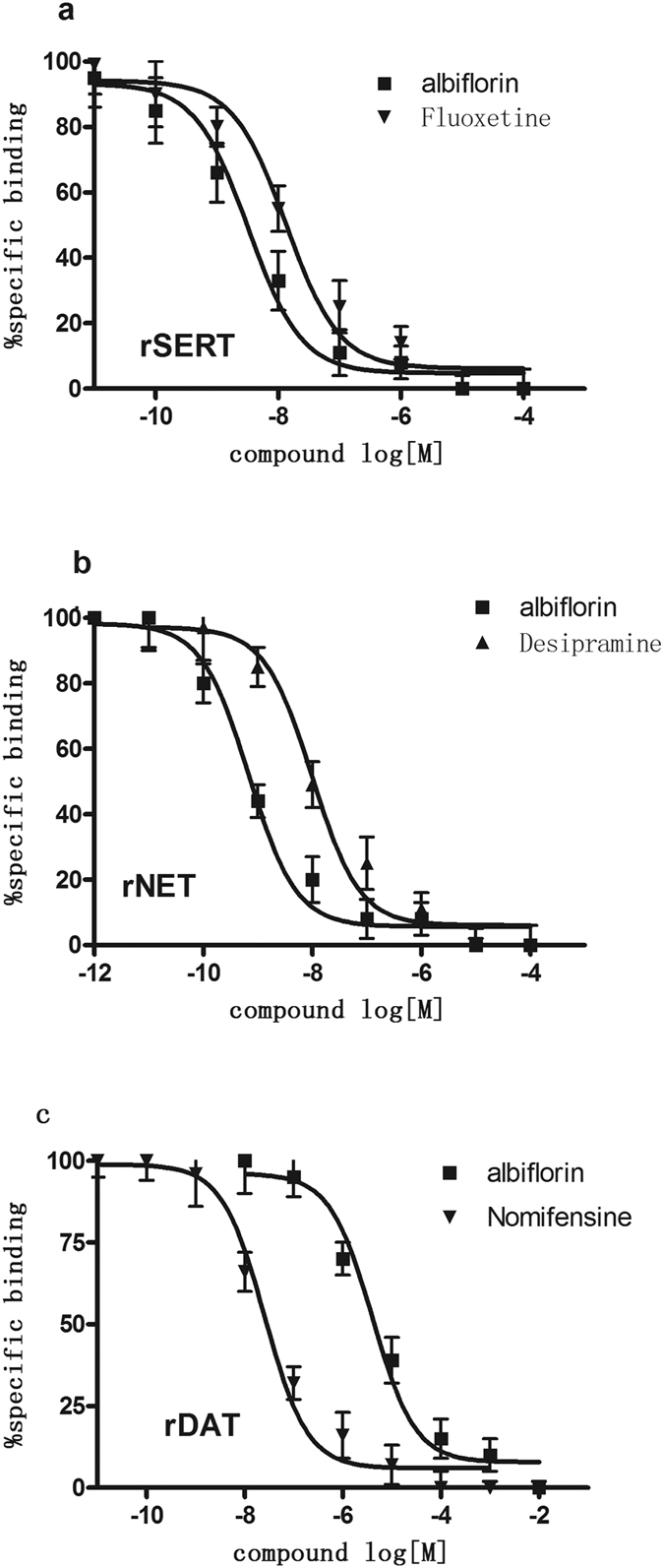
Albiflorin competes for the binding of radioligands specific to the rSERT (**a**) rNET (**b**) and rDAT (**c**). For each transporter bioassay, a known comparator was used [fluoxetine (5-HT reuptake inhibitor); desipramine (NE reuptake inhibitor); and nomifensine (DA reuptake inhibitor)]. The IC_50_ value was generated from each of these curves and used to generate the K_i_ values. Each data point depicted represents the mean ± S.E.M. of three independent experiments performed in triplicate. The K_i_ values for albiflorin and the comparators are shown in Table 1.

To evaluate the ability of albiflorin to bind to monoamine transporters, competition binding assays were conducted using rat brain tissues. We found that albiflorin showed a high affinity for the rat SERT and NET (Table 1). For SERT, the binding of [^3^H]-citalopram to rat cerebral cortical membranes was competitively inhibited by albiflorin and fluoxetine. Our results showed that albiflorin was 3-fold more potent than fluoxetine in binding to the rat SERT (K_i_ values for albiflorin: 5.25 ± 0.17; fluoxetine: 16.32 ± 1.02 nM; Table 1; Fig. 1a). The affinity of albiflorin to the rat NET was 6-fold more potent than that for desipramine, a known specific inhibitor of NET. The K_i_ values for albiflorin and desipramine were 1.25 ± 0.77 and 7.38 ± 0.67 nM, respectively (Table 1; Fig. 1b). To evaluate the ability of albiflorin to bind to DAT, we added [^3^H]-WIN35,428 (1.0 nM) to the brain membrane preparation and used albiflorin or the DAT-specific ligand nomifensine as the competitive compounds. We found that the affinity of albiflorin at the rat DAT was much lower than that for nomifensine (K_i_ values for albiflorin and nomifensine were 3,656 ± 25.36 and 29.36 ± 2.36 nM, respectively; Table 1; Fig. 1c). Comparing the affinity of albiflorin to the three monoamine transporters, we found that albiflorin binds to SERT and NET with the highest affinity.

### Binding of albiflorin to human monoamine transporters

**Figure 2:**
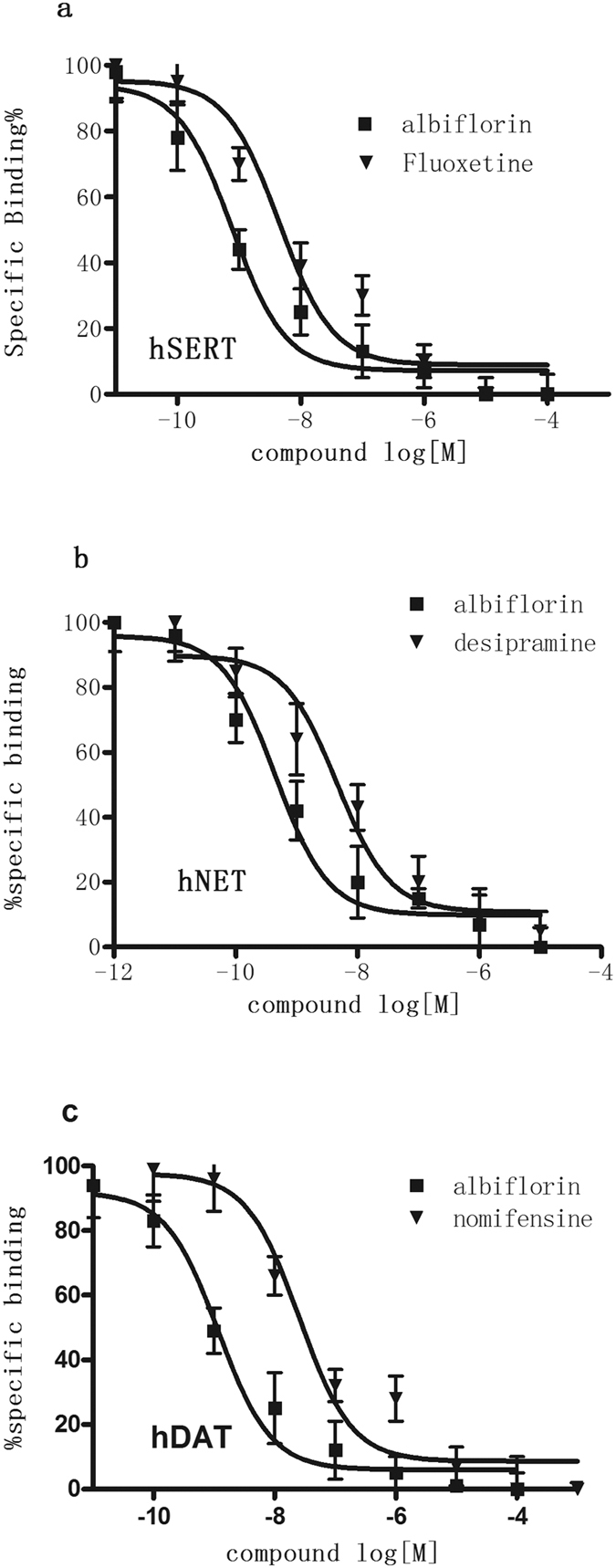
Albiflorin competes for the binding of radioligands specific to the cloned human SERT (**a**) NET (**b**) and DAT (**c**) expressed in HEK293 cell lines. For each transporter bioassay, a known comparator was used [fluoxetine (5-HT reuptake inhibitor); desipramine (NE reuptake inhibitor); and nomifensine (DA reuptake inhibitor)]. The IC_50_ value was generated from each of these curves and used to generate the Ki values. Each data point depicted represents the mean ± S.E.M. of three independent experiments performed in triplicate. The K_i_ values for albiflorin and the comparators are shown in Table 1.

To determine whether albiflorin has affinities to the human monoamine transporters similar to those in the rat, we used HEK293 cells transfected with SERT, NET, or DAT and tested the ability of albiflorin to compete with specific inhibitors of these different transporters. As shown in Table 1, albiflorin showed the highest affinities to the human SERT and NET, consistent with our results from rats. Albiflorin and fluoxetine dose-dependently competed against the binding of the SERT radioligand [^3^H]-citalopram in membranes from cells transfected with hSERT. The K_i_ values for albiflorin and fluoxetine were 6.45 ± 0.48 and 14.23 ± 1.25 nM, respectively (Table 1; Fig. 2a). Albiflorin and desipramine competed against the binding of radiolabeled [^3^H]-nisoxetine in membranes from cells transfected with hNET, with K_i_ values of 1.23 ± 0.07 and 6.35 ± 0.54 nM, respectively (Table 1; Fig. 2b). Albiflorin and nomifensine competed against the binding of [^3^H]-WIN35,428 at the hDAT, with K_i_ values of 1478 ± 33.25 and 14.36 ± 1.02 nM, respectively (Table 1; Fig. 2c). Similar to the rat monoamine transporter, the affinity of albiflorin for hSERT and hNET was higher than that for hDAT.

### Albiflorin inhibits monoamine uptake in rat synaptosomes

**Figure 3:**
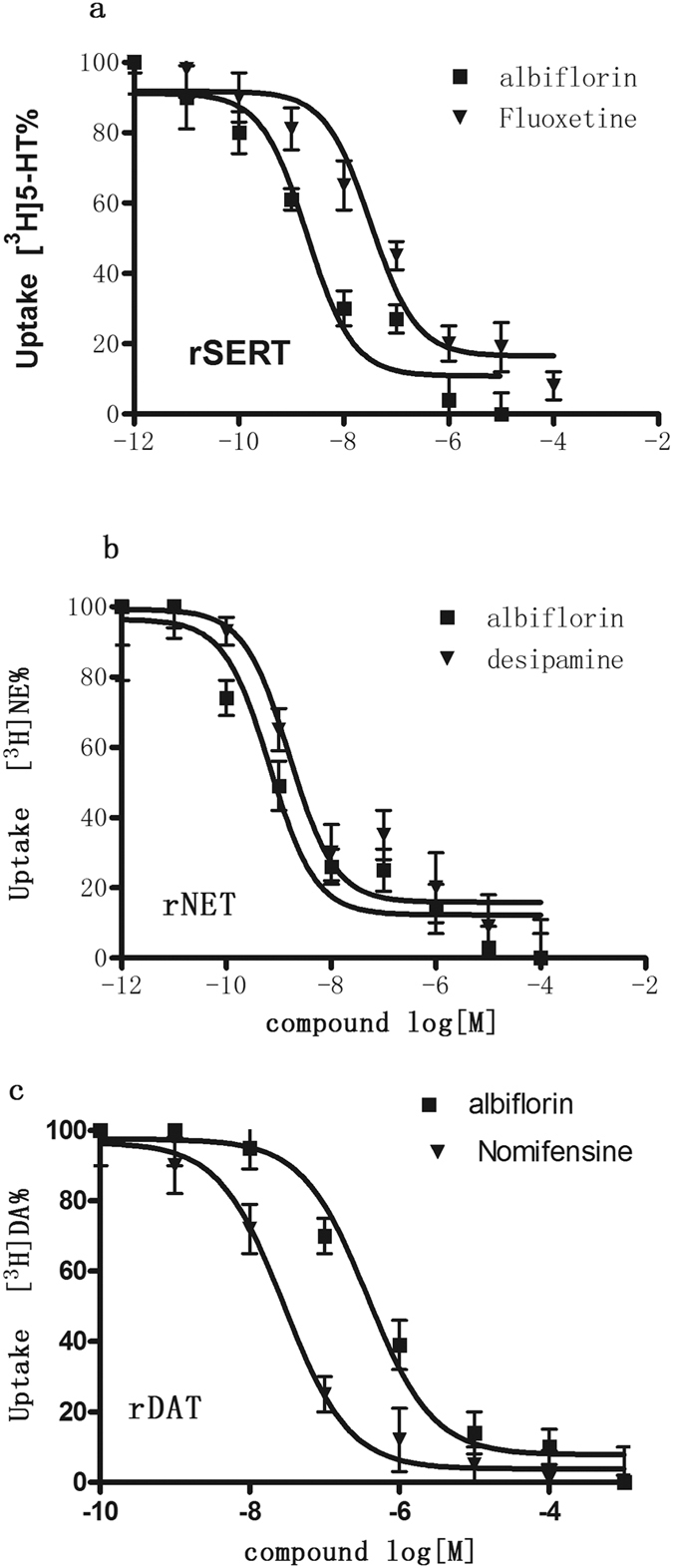
Functional activity of albiflorin demonstrates inhibition of radiolabeled uptake of serotonin (**a**) norepinephrine (**b**) and dopamine (**c**) in rat synaptosomes. For each uptake transporter bioassay, a known comparator was used [fluoxetine (5-HT reuptake inhibitor); desipramine (NE reuptake inhibitor); nomifensine (DA reuptake inhibitor)]. Each data point depicted represents the mean ± S.E.M. of three independent experiments performed in triplicate. The IC_50_values for albiflorin are shown in Table 2.

To examine the effect of albiflorin on monoamine transport activity, rat brain synaptosomes were prepared and treated with various concentrations of albiflorin. Our results showed that albiflorin dose-dependently inhibited all three transporters (SERT, DAT and NET; Fig. 3a–c). The K_i_ values for uptake of [^3^H]-5-HT into rat cerebral cortical synaptosomes was 3.04 ± 0.14 and 52.36 ± 2.36 nM for albiflorin and fluoxetine, respectively (Table 2; Fig. 3a). Albiflorin and desipramine inhibited uptake of [^3^H]-NE into hypothalamic synaptosomes with K_i_ values of 0.98 ± 0.24 and 2.35 ± 0.28 nM, respectively (Table 2; Fig. 3b). Albiflorin hardly inhibited [^3^H]-DA uptake in rat striatal synaptosomes, with K_i_ values of 3,689 ± 114 and 35.36 ± 2.21 nM for albiflorin and nomifensine, respectively (Table 2; Fig. 3c). Thus, consistent with its binding profile, albiflorin exhibited more potent inhibition of 5-HT and NE uptake at SERT and NET, respectively, than their corresponding selective inhibitors, but was less effective at inhibiting DA uptake at DAT (Table 2).

### Albiflorin inhibits uptake activity of human monoamine transporters

**Figure 4:**
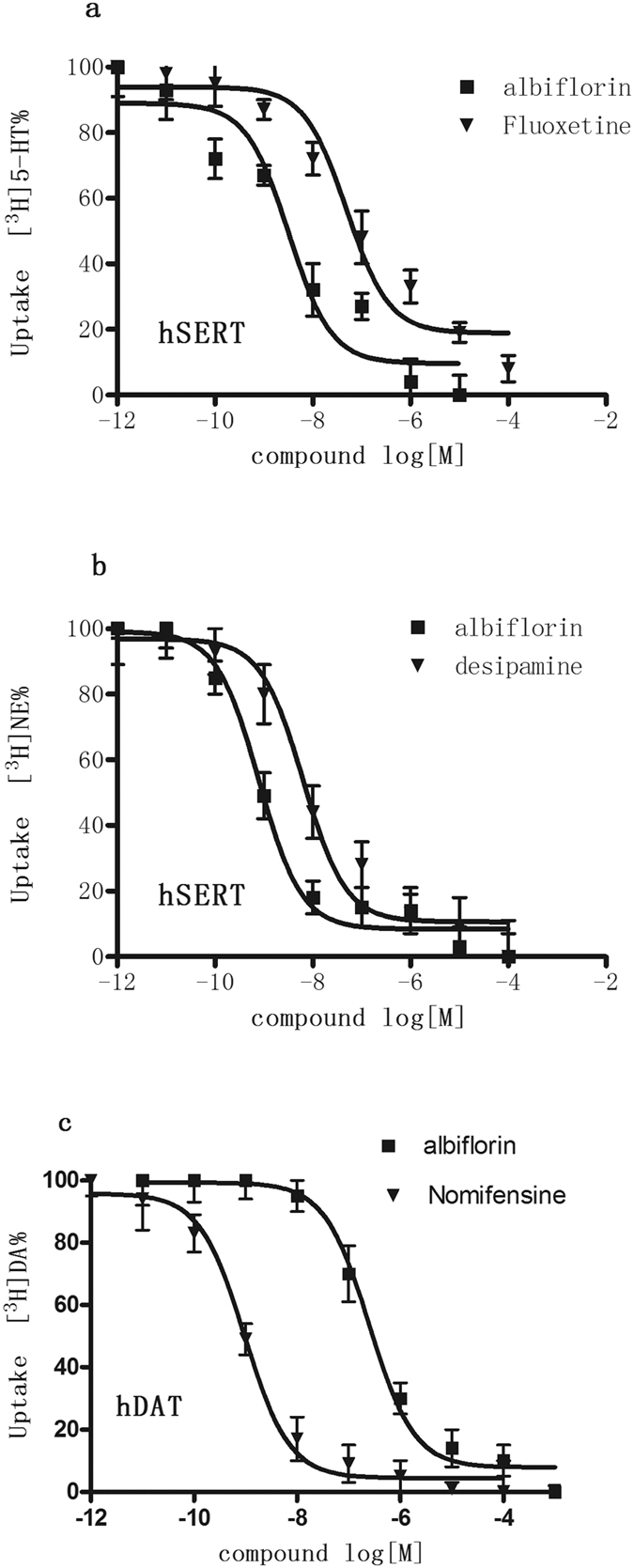
Functional activity of albiflorin demonstrates inhibition of radiolabeled uptake of serotonin (**a**) norepinephrine (**b**) and dopamine (**c**) in cloned human transporters expressing in HEK293 cell lines. For each uptake transporter bioassay, a known comparator was used [fluoxetine (5-HT reuptake inhibitor); desipramine (NE reuptake inhibitor); nomifensine (DA reuptake inhibitor)]. Each data point depicted represents the mean ± S.E.M. of three independent experiments performed in triplicate. The IC_50_ values for albiflorin are shown in Table 2.

HEK293 cells were transfected with hSERT, hDAT, or hNET and were treated with albiflorin at various concentrations. Consistent with the results from rat synaptosomes, we found that albiflorin also significantly inhibited the activities of NET and SERT in these transfected cell lines. The K_i_ values for albiflorin to inhibit the uptake of [^3^H]-5-HT, [^3^H]-5-NE and [^3^H]-DA into cells expressing the corresponding human recombinant transporters were 2.36 ± 0.24 nM, 0.36 ± 0.08 nM and 2236 ± 25 nM, respectively (Table 2; Fig. 4). These data demonstrate that albiflorin has a higher potency for inhibiting the transporter activities of SERT and NET than it does for DAT.

### Albiflorin selectively binds to NET and SERT

To determine whether albiflorin might also bind to other potential targets, we tested the ability of albiflorin (10 μM) to bind to the following protein targets, which included G-protein coupled receptors: D_1_, D_2_, D_3_, D_4_, D_5_, 5-HT_1A_, 5-HT_1B_, 5-HT_1D_, 5-HT_2a_, 5-HT_2C_, 5-HT_3_, 5-HT_5A_, 5-HT_6_, 5-HT_7_, α_1A_, α_1B_, α_2A_, β_1_, M_1_, M_2_, M_3_, M_4_, M_5_, μ, κ, δ, A_1_, A_2A_, H_1_, H_2_, H_3_, H_4_. As shown in Table 3, albiflorin showed no significant affinity for any of these tested targets, suggesting that the binding of albiflorin is selective for SERT and NET.

### Albiflorin administration induces an increase in monoamine levels in the microdialysate from the rat hypothalamus *in vivo*

**Figure 5:**
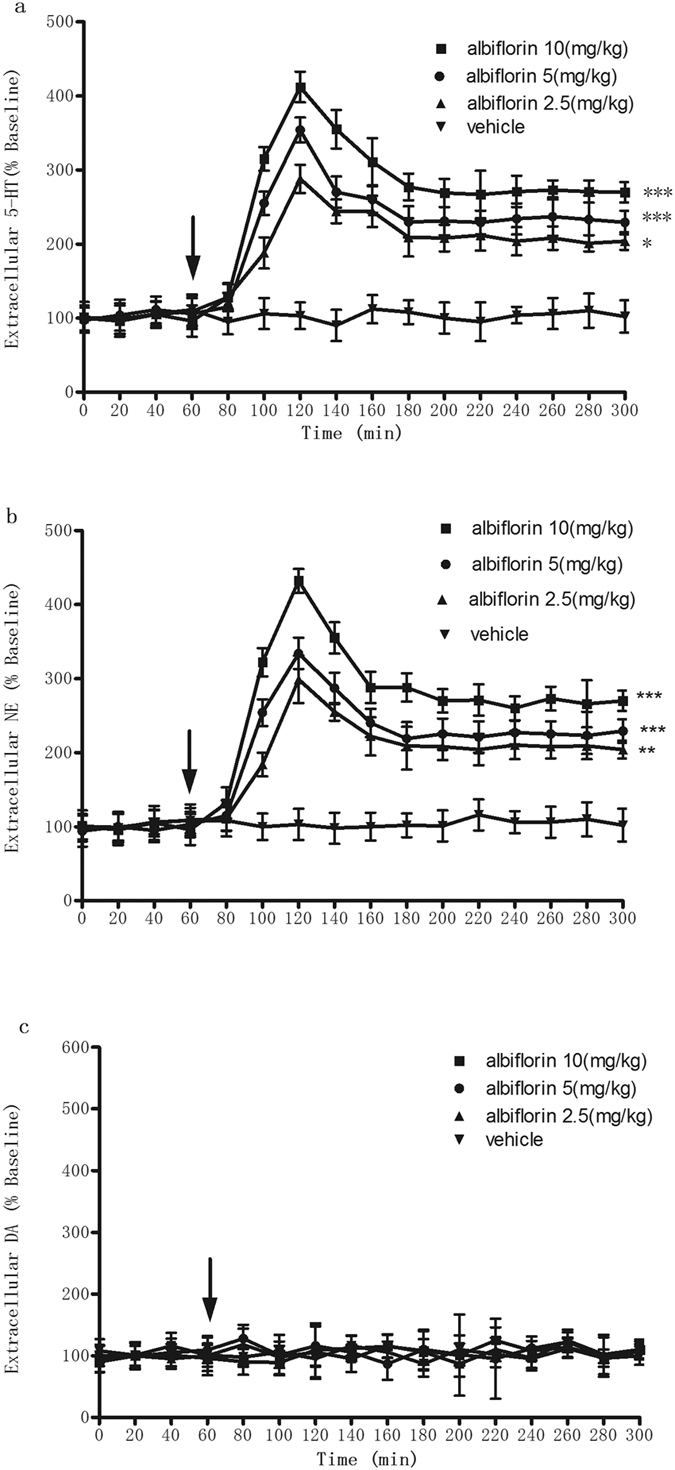
Effect of albiflorin on extracellular 5-HT, NE and DA levels in the frontal cortex of rats measured by micro dialysis (n = 6–9 per group). Data points represent mean ± S.E.M. of 5-HT (**a**), NE (**b**) and DA (**c**) levels expressed relative to basal pretreatment values (define as 100%). Arrow indicates time of drug administration. albiflorin was administered via oral gavage in a volume of 1 ml/kg to rats. *P < 0.05 indicate significant differences compared with saline.

To evaluate the effect of albiflorin on central extracellular monoamine levels, we administered albiflorin orally to rats and determined monoamine levels using *in vivo* microdialysis. We found that acute administration of albiflorin led to a significant increase in hypothalamic 5-HT levels in dose- and time-dependent manners (two-way repeated-measures analysis of variance [ANOVA]: treatment, F_(3,29)_ = 24.0, P < 0.0001; time, F_(3,428)_ = 69.70, P < 0.0001). Post hoc Tukey’s tests showed that, compared with vehicle, albiflorin above concentrations of 2.5 mg/kg increased 5-HT at all times examined, peaking 1 h post albiflorin administration. At this peak time (1 h after treatment), the maximal effect of albiflorin was observed at a dose of 10 mg/kg (Fig. 5a). Similarly, acute administration of albiflorin induced a dose- and time-dependent increase in hypothalamic NE levels, peaking 1 h after albiflorin administration of 10 mg/kg (treatment: F_(3,25)_ = 21.280, P < 0.0001; time: F_(3,329)_ = 74.35, P < 0.0001). Post hoc Tukey’s tests showed that all doses, including 3.5 mg/kg of albiflorin, induced increases in NE levels at all times examined (Fig. 5b). By contrast, DA levels in the hypothalamus were not elevated after administration of albiflorin (treatment: F_(3,29)_ = 6.07, P > 0.05; time: F_(3,345)_ = 11.75, P > 0.05; Fig. 5c). Post hoc Tukey’s tests showed that at doses of 3.5, 7.0 and 14.0 mg/kg, albiflorin induced no increase in DA levels at any time evaluated (Fig. 5c).

## Discussion

Despite the wide use of Radix paeoniae Alba for treating mood disorders, there is a lack of scientific reports evaluating its pharmacological effects at the level of the receptor. In the present study, we found that albiflorin bound to NET and SERT with high affinity in both a rat synaptosomal preparation and intact cell lines expressing human transporters. Albiflorin showed higher affinity for SERT than for NET, whereas it showed only weak affinity for DAT. In addition, albiflorin (10 μM) demonstrated no significant activity at numerous nontransporter targets. The results of *in vitro* bioassay experiments show that albiflorin exhibited selective inhibitory activity for neurotransmitter uptake at the 5-HT and NE monoamine transporters and increased extracellular levels of NE and 5-HT when compared with baseline levels of monoamines in the hypothalamus of rats. Taken together, these data support the view that albiflorin is a novel serotonin and norepinephrine reuptake inhibitor.

In our present study, we conducted assays using both rat brain synaptosomes and a human transporter-transfected cell line to evaluate the effects of albiflorin and to clarify whether a species difference occurred. Previous studies have reported conflicting results regarding the affinities of SSRIs, including citalopram, paroxetine and fluoxetine, at rat and human SERTs^9^,^10^,^11^. These studies highlighted the importance of determining drug affinities in both species^12^. Our results demonstrated that albiflorin potently interacts with SERT and NET in both species. We found that albiflorin demonstrated equivalent and high affinity for both rat and human SERTs and showed a 17-fold higher affinity than fluoxetine in both rat SERT and human SERT binding assays. In addition, albiflorin potently bound hNET and rat NET with high affinity, showing a 4-fold higher affinity than desipramine in rat NET and hNET binding assays. Consistent with these results from our binding assays, the data from our functional studies exhibited a similar pattern in the ability of albiflorin to inhibit monoamine reuptake in rat synaptosomes and intact cells expressing human transporters. Albiflorin was approximately 10-fold more potent at inhibiting NE uptake than 5-HT uptake. Furthermore, assays examining the competition of albiflorin for the binding of [^3^H]-WIN35,428, a selective DA reuptake inhibitor, showed inhibitory activity of albiflorin at only high micromolar concentrations. Together, our results indicate that this relatively weak binding interaction would produce no functional consequence on dopamine levels at the concentrations required to inhibit NET and SERT.

Nonselective interaction of uptake inhibitors with neuronal receptors may increase the potential of adverse effects. Previous studies have shown that citalopram and clomipramine displayed antagonist properties with modest affinities at 5-HT_2C_ sites^13^,^14^. Reboxetine was also found to have mild affinity for h5-HT_2C_ receptors. Although blockade of 5-HT_2C_ sites favourably influences mood, it may also elicit hyperphagia^15^. Moreover, antagonism by clomipramine of histamine H_1_ receptors contributes to weight gain, and the actions of clomipramine on H_1_ and acetylcholine muscarinic M_1_ receptors cause cardiovascular autonomic and sedative side effects^10^. In our current studies, we screened a series of potential targets that albiflorin might interact with, but albiflorin displayed no or very weak affinity to all of them, including in particular, receptors for 5-HT, NE, DA, histamine and muscarine, suggesting that albiflorin may be a novel serotonin and norepinephrine reuptake inhibitor.

In support of the results from our *in vitro* studies, the results of our *in vivo* microdialysis studies showed that the administration of albiflorin significantly increased the levels of NE and 5-HT in the hypothalamus of rats at doses (3.5, 7.0 and 14.0 mg/kg) that were effective for antidepressant-like actions in the mouse forced-swim and tail-suspension tests^4^. These results suggested that the elevated extracellular monoamines were the consequence of functional blockade of the monoamine transporters. The increases in monoamine levels were observed 60 min after administration of albiflorin, consistent with the results of the forced-swim and tail-suspension tests.

Given the complicated etiology of depression, the important role of 5-HT and NE in antidepressant therapy, and the crosstalk between these neurotransmitters, it has been suggested that drugs that simultaneously increase the levels of 5-HT and NE may exert increased therapeutic efficacy^11^,^15^. Increasing 5-HT and NE levels in the local brain environment may result in increased levels of these key neurotransmitters in the circulation. These transmitters play important roles in the peripheral nervous system, as well as in the periphery^16^, and thus may have utility both centrally and peripherally in treating disorders associated with modified levels of 5-HT and NE, such as those involving various pain modalities^17^,^18^, inflammatory processes^19^,^20^ and urinary incontinence^21^. Compounds with dual-acting 5-HT and NE monoamine transporter inhibition have been shown to have multiple applications^22^,^23^. Therefore, as a novel 5-HT and NE reuptake inhibitor, the utility and precise functional activities of albiflorin warrant further study.

## Methods

### Drugs and reagents

Albiflorin, obtained from Beijing Wonner Biotech Co., Ltd. (China), has a molecular formula of C_23_H_28_O_11_ and a molecular weight of 480.46. [^3^H]-citalopram, [^3^H]-WIN35,428, [^3^H]-nisoxetine, [^3^H]5-HT, [^3^H]-NE and [^3^H]-DA were purchased from PerkinElmer Life Sciences (NEN, Boston, MA). Scintillation cocktail (Ultima Gold, catalogue number 6013329) was purchased from PerkinElmer Life and Analytical Sciences. Dulbecco’s modified Eagle’s medium and foetal bovine serum were purchased from Invitrogen Inc. (Grand Island, NY) and HyClone Corp. (South Logan, UT), respectively. Desipramine (catalogue number D-3900), fluoxetine (catalogue number F-132), paroxetine (catalogue number P-1372) and nomifensine (catalogue number M-2017) were purchased from Sigma-Aldrich (St. Louis, MO). All other radioligands were purchased from PerkinElmer Life and Analytical Sciences.

### Animals

Male Sprague–Dawley rats (180–200 g) were obtained from Beijing Vital River Laboratory Animal Technology Company, Beijing, China, and were acclimated to the facility for 1 week before use in the experiments. The animals were housed at 22 ± 1 °C with a 12-h light/dark cycle (lights on at 7:00 am) and were given ad libitum access to water and food. All experiments were performed in accordance with relevant guidelines and regulations approved by the Experimental Animal Research Committee of Capital Medical University.

### Rat monoamine transporter binding assay

Male Sprague–Dawley rats were killed by cervical dislocation, and their brains were rapidly removed and then dissected on ice. The cerebral cortex, hypothalamus and striatum were homogenised in 4 mL of assay buffer (50 mM Tris–HCl buffer, pH 7.4.) and centrifuged at 40,000× *g* for 10 min at 4 °C. The pellets were centrifuged twice at 20,000× *g* and then resuspended in 4 mL of assay buffer. The samples were incubated at 37 °C for 20 min to remove endogenous 5-HT, followed by two centrifugations, as previously described by Wong *et al.* (1993b). Bio-Rad protein assay reagents (1:5 ratio) were used to analyse the protein concentration of the synaptosomal suspension. The membrane samples (containing 50 μg protein per sample) were incubated with various concentrations of drugs, including [^3^H]-citalopram (1.2 nM, for SERT), [^3^H]-nisoxetine (1.0 nM, for NET) or [^3^H]-WIN35,428 (1.0 nM, for DAT) in 50 mM Tris-HCl, pH 7.4, 150 mM NaCl (300 mM NaCl for nisoxetine). Nonspecific binding was determined by including separate samples of 1 μM fluoxetine, 10 μM desipramine and 10 μM nomifensine for SERT, NET and DAT, respectively.

### Human monoamine transporter binding assay

Membranes were prepared from HEK293 cells stably expressing hSERT, hNET, or hDAT as described previously^24^,^25^. Briefly, [^3^H]-citalopram (1 nM), [^3^H]-nisoxetine (2 nM), or [^3^H]-WIN35,428 (2 nM) was used to label human 5-HT, NE, or DA transporters, respectively. Nonspecific binding was determined by including separate samples of 1 μM fluoxetine, 10 μM desipramine, or 10 μM nomifensine for SERT, NET and DAT, respectively.

### Other potential binding targets

Albiflorin (10 μM) was tested for numerous other potential binding targets by using standard receptor binding procedures^24^,^25^. The tested targets included the following receptors: D_1_, D_2_, D_3_, D_4_, D_5_, 5-HT_1A_, 5-HT_1B_, 5-HT_1D_, 5-HT_2a_, 5-HT_2C_, 5-HT_3_, 5-HT_5A_, 5-HT_6_, 5-HT_7_, α_1A_, α_1B_, α_2A_, β_1_, M_1_, M_2_, M_3_, M_4_, M_5_, μ, κ, δ, A_1_, A_2A_, H_1_, H_2_, H_3_, H_4_.

### Rat synaptosome uptake assay

Male Sprague–Dawley rats were killed by asphyxiation with carbon dioxide and cervical dislocation followed by decapitation. The brains were removed quickly and the cerebral cortex, hypothalamus and striatum were dissected on ice. All dissected tissues were homogenised using a Teflon-glass tissue homogeniser in 4 mL of ice-cold sucrose (0.32 M). The homogenised tissue was centrifuged at 2,000× *g* for 10 min at 4 °C. The synaptosomal pellets were resuspended in 8 mL of ice-cold assay buffer (10 mM HEPES, 133 mM NaCl, 4.85 mM KCl, 1.2 mM KH_2_PO_4_, 1.5 mM MgSO_4_, 1.5 mM CaCl_2_, 11.1 mM glucose, pH 7.4). Bio-Rad protein assay reagents (1:5 ratio) were used to analyse the protein concentration of the synaptosomal suspension. Uptake of 5-HT, NE and DA was determined as described previously^9^,^24^,^25^. The compounds to be tested were dissolved in DMSO, further diluted with assay buffer, and tested over a 10-log unit concentration range in duplicate. Samples of 30–50 μg of synaptosomes (per sample in a total volume of 200 μL) were preincubated at 37 °C in a shaking water bath for 10 min. Vehicle or modifiers were then added for 10–20 min. Uptake was initiated by adding [^3^H]-5-HT (20 nM), [^3^H]-NE (20 nM), or [^3^H]-DA (50 nM). After a 10-min incubation at 37 °C, uptake was terminated by filtration through (polyethyleneimine-coated) GF/B Whatman filters using a Brandel Cell Harvester (Brandel, Gaithersburg, MD). Nonspecific uptakes for DAT, SERT and NET were determined using 10 μM nomifensine, 10 μM citalopram and 10 μM desipramine, respectively.

### Transporter-transfected cell lines

The uptake of 5-HT, NE and DA into HEK293 cells stably expressing human transporters was determined using methods previously described with slight modifications^26^,^27^. Briefly, the culture medium was removed from cells, and the cells were washed once with phosphate-buffered saline. Krebs-Ringers-HEPES (KRH) assay buffer (100 μL/well, containing 120 mM NaCl, 4.7 mM KCl, 2.2 mM CaCl_2_, 1.2 mM MgSO_4_, 1.2 mM KH_2_MgSO_4_, 10 mM HEPES, pH 7.4) was then added. Various concentrations of drugs were administered to cells in a 24-well plate. The uptake was initiated by the addition of [^3^H]-5-HT (50 nM), [^3^H]-NE (80 nM), or [^3^H]-DA (50 nM). Uptake was terminated by five washes using cold KRH assay buffer after 10-min incubation at 37 °C. The cells were solubilised using 400 μL of scintillation fluid and counted using a Packard Top Count scintillation counter.

### *In vivo* microdialysis studies

The effect of albiflorin on extracellular monoamine levels in frontal cortices was determined using methods previously described^1^. Microdialysis probes (CMA Microdialysis) with an active membrane surface of 2 mm were perfused with artificial cerebrospinal fluid (125 mM NaCl, 3 mM KCl, 0.75 mM MgSO_4_ and 1.2 mM CaCl_2_, pH 7.4) *in situ* for at least 18 h before experimentation. The microdialysis probe was then implanted via the guide cannula into the hypothalamus and perfused with artificial cerebral spinal fluid at a flow rate of 1 μL/min. A 3-h stabilisation period was allowed after probe implantation, and dialysate sampling was conducted according to previously described methods^1^,^28^. Briefly, five control samples (ficomole/20-μL sample) were obtained prior to drug injection to demonstrate a steady baseline. All subsequent values were expressed as a percentage of the mean preinjection value (i.e., percentage of baseline). After baseline sampling was completed, animals were treated with albiflorin (3.5, 7.0, 14.0 mg/kg) or vehicle (0.2% Tween 80 and 0.5% methylcellulose dissolved in water) orally administered at the end of the sixth sample collection. For a minimum of 3 h after albiflorin administration, dialysis samples were collected every 20 min and analysed to determine the levels of NE, 5-HT and DA. At the end of each experiment, animals were euthanised, and the probe placement was verified by histology. Data from rats with incorrect probe placement were discarded. A 20-μL sample from the hypothalamus was analysed for 5-HT, NE and DA content using methods described previously^1^,^28^. The HPLC system consisted of a microbore reverse-phase column (particle size 5 μm, 150 × 4.6 mm; Model C-18, DIKMA Technologies Ltd., Beijing, China), an Agilent 1100 pump (flow rate 1.0 mL/min; Agilent Technologies, Palo Alto, CA, USA) and a Hewlett-Packard HP 1049A glassy carbon amperometric detector (Agilent Technologies, Palo Alto, CA, USA). The mobile phase consisted of 85 mM citrate, 100 mM sodium acetate, 0.9 mM octyl sodium sulphate, 0.2 mM EDTA and 15% methanol at pH 3.7. External standard curves were used to quantify the amounts of NE, 5-HT and DA in each sample, which was calculated using the area under the curve.

### Statistical analysis

All data for inhibition curves were analysed by nonlinear regression using Prism software (GraphPad Software Inc., San Diego, CA) to determine IC_50_ values. For the microdialysis experiments, two-way repeated-measures ANOVA followed by Tukey’s test was used to compare the percentage increase over baseline between groups, time and treatment as independent factors, and time as the repeated factor.

## Additional Information

**How to cite this article**: Jin, Z. *et al.* Receptor and transporter binding and activity profiles of albiflorin extracted from *Radix paeoniae Alba. Sci. Rep.* **6**, 33793; doi: 10.1038/srep33793 (2016).

## Figures and Tables

**Table 1:** Binding affinity of albiflorin at monoamine transporters.

	Species	Radioligand	Tissues	K_i_ values (nM)	
				albiflorin	Comparator
SERT	Rat	[^3^H]-citalopram	Cerebral cortex	5.25 ± 0.17	16.32 ± 1.02 (fluoxetine)
SERT	Human	[^3^H]-citalopram	HEK293 cell line	6.45 ± 0.48	14.23 ± 1.25 (fluoxetine)
NET	Rat	[^3^H]-nisoxetine	Hypothalamus	1.25 ± 0.77	7.38 ± 0.67 (desipramine)
NET	Human	[^3^H]-nisoxetine	HEK293 cell line	1.23 ± 0.07	6.35 ± 0.54 (desipramine)
DAT	Rat	[^3^H]-win35,428	Striatum	3656 ± 25.36	29.36 ± 2.36 (nomifensine)
DAT	Human	[^3^H]-win35,428	HEK293 cell line	1478 ± 33.25	14.36 ± 1.02 (nomifensine)

K_i_ value = IC_50_/(1 + L/K_D_), where L = equals concentration of radioligand added. Comparators are fluoxetine (selective serotonin reuptake inhibitor), desipramine (norepinephrine reuptake inhibitor), and nomifensine (dopamine reuptake inhibitor). Data presented were generated in a minimum of two separate assays conducted on different test days. Each test concentration was run in triplicate and presented as mean ± S.E.M.

**Table 2:** Influence of albiflorin at rat cerebral synaptosome or intact cell expressing human transporters uptake of [^3^H]serotonin(5-HT), [^3^H]norepinephrine (NE), and [^3^H]dopamine (DA).

	Species	Radioligand	Tissues	IC_50_values(nM)	
				albiflorin	Comparator
SERT	Rat	[^3^H]-5-HT	Mid brain	3.04 ± 0.84	52.36 ± 2.36 (fluoxetine)
SERT	Human	[^3^H]-5-HT	HEK293 cell line	2.36 ± 0.74	34.25 ± 2.33 (fluoxetine)
NET	Rat	[^3^H]-NE	Frontal cortex	0.98 ± 0.24	2.35 ± 0.28 (desipramine)
NET	Human	[^3^H]-NE	HEK293 cell line	0.36 ± 0.08	1.22 ± 0.38 (desipramine)
DAT	Rat	[^3^H]-DA	Striatum	3689 ± 114	35.36 ± 2.21 (nomifensine)
DAT	Human	[^3^H]-DA	HEK293 cell line	2236 ± 25	18.36 ± 2.34 (nomifensine)

Data IC_50_ values are presented as mean ± S.E.M. IC_50_ of three independent determinations, each performed in triplicate. Data presented were generated in a minimum of two separate assays conducted on different test days.

**Table 3:** Binding affinity of albiflorin for CNS receptors.

Targets	Species	Tissue	Radioligand(nM)	% Inhibition of binding at 10 μM albiflorin	IC_50_ values for albiflorin (nM)
D_1_	Human	HEK293 cell line	[^3^H]-SCH23390(1)	54.20%	2989 ± 166
D_3_	Human	CHO cell lines	[^3^H]-spiperone(1)	21.30%	3655 ± 212
D_2_	Human	HEK293 cell line	[^3^H]-spiperone(1)	12.50%	>10,000
D_4_	Human	HEK293 cell line	[^3^H]-spiperone(1)	19.50%	>10,000
D_5_	Human	HEK293 cell line	[^3^H]-SCH23390(1)	32.50%	>10,000
5-HT_1A_	Human	HEK293 cell line	[^3^H]-8-OH-DPAT(2)	69.60%	3999 ± 662
5-HT_1B_	Human	HEK293 cell line	[^3^H]-LSD(6)	16.60%	>10,000
5-HT_1D_	Human	HEK293 cell line	[^3^H]-LSD(10)	22.36%	>10,000
5-HT_2a_	Human	HEK293 cell line	[^3^H]-ketanserin(1)	55.36%	4005 ± 113
5-HT_2C_	Human	CHO cell lines	[^3^H]-mesulergine(1)	33.66%	>10,000
5-HT_3_	Human	HEK293 cell line	[^3^H]-BRL3694	17.38%	>10,000
5-HT_5A_	Human	HEK293 cell line	[^3^H]-LSD(1)	20.11%	>10,000
5-HT_6_	Human	HEK293 cell line	[^3^H]-LSD(1)	22.36%	>10,000
5-HT_7_	Human	HEK293 cell line	[^3^H]-LSD(4)	14.55%	>10,000
α_1A_	Rat	Frontal cortex	[^3^H]-parzosin(1)	56.33%	4705 ± 113
α_1B_	Human	HEK293 cell line	[^3^H]-parzosin(1)	12.33%	>10,000
α_2A_	Rat	Cortex	[^3^H]-clonidine(2)	36.66%	>10,000
β_1_	Human	HEK293 cell line	[^3^H]-DHA(1)	63.22%	3355 ± 212
M_1_	Human	CHO cell line	[^3^H]-NMS(1)	21.28%	>10,000
M_2_	Human	HEK293 cell line	[^3^H]-NMS(1)	13.33%	>10,000
M_3_	Human	CHO cell line	[^3^H]-NMS(1)	25.66%	>10,000
M_4_	Human	CHO cell line	[^3^H]-NMS(1)	15.33%	>10,000
M_5_	Human	CHO cell line	[^3^H]-NMS(1)	20.11%	>10,000
μ	Human	CHO cell line	[^3^H]-dipnorphine(1)	23.66%	>10,000
κ	Human	CHO cell line	[^3^H]-dipnorphine(1)	32.33%	>10,000
δ	Human	CHO cell line	[^3^H]-dipnorphine(1)	66.33%	3655 ± 201
A_1_	Human	CHO cell line	[^3^H]-DPCPX(1)	21.23%	>10,000
A_2A_	Human	HEK293 cell line	[^3^H]-CGS21680(10)	3.323%	>10,000
H_1_	Human	CHO cell line	[^3^H]-pyrilamine(2)	6.33%	>10,000
H_2_	Human	HEK293 cell line	[^3^H]-tiotodine(1)	68.99%	4005 ± 201
H_3_	Human	CHO cell line	[^3^H]-hestamine(1)	12.33%	>10,000
H_4_	Human	CHO cell line	[^3^H]-histamine(1)	6.33%	>10,000

The concentration of albiflorin for primary binding experiments was 10 μM, and % inhibition (mean of 4 determinations) is shown. Compound exhibiting >50% inhibition at any receptor was evaluated in secondary binding assays with full concentration curves to derive IC_50_ (mean ± S.E.M. n = 3).
